# Sphingolipids in Childhood Asthma and Obesity (SOAP Study): A Protocol of a Cross-Sectional Study

**DOI:** 10.3390/metabo13111146

**Published:** 2023-11-11

**Authors:** Belavendra Antonisamy, Harshita Shailesh, Yahya Hani, Lina Hayati M. Ahmed, Safa Noor, Salma Yahya Ahmed, Mohamed Alfaki, Abidan Muhayimana, Shana Sunny Jacob, Saroja Kotegar Balayya, Oleksandr Soloviov, Li Liu, Lisa Sara Mathew, Kun Wang, Sara Tomei, Alia Al Massih, Rebecca Mathew, Mohammed Yousuf Karim, Manjunath Ramanjaneya, Stefan Worgall, Ibrahim A. Janahi

**Affiliations:** 1 Department of Pediatric Medicine, Sidra Medicine, Doha P.O. Box 26999, Qatar; abelavendra@sidra.org (B.A.); hshailesh@sidra.org (H.S.); yhani1@sidra.org (Y.H.); lhasabelgawi@sidra.org (L.H.M.A.); snoor@sidra.org (S.N.); sahmed10@sidra.org (S.Y.A.); malfaki@sidra.org (M.A.); amuhayimana@sidra.org (A.M.); 2 Analytical Chemistry Core, Advanced Diagnostic Core Facilities, Sidra Medicine, Doha P.O. Box 26999, Qatar; sjacob@sidra.org (S.S.J.); skotegarbalayya@sidra.org (S.K.B.); 3 Clinical Genomics Laboratory, Integrated Genomics Services, Sidra Medicine, Doha P.O. Box 26999, Qatar; osoloviov@sidra.org (O.S.); lliu@sidra.org (L.L.); lmathew@sidra.org (L.S.M.); kwang@sidra.org (K.W.); 4 Omics Core, Integrated Genomics Services, Sidra Medicine, Doha P.O. Box 26999, Qatar; stomei@sidra.org (S.T.); aalmassih1@sidra.org (A.A.M.); rmathew1@sidra.org (R.M.); 5 Department of Pathology, Sidra Medicine, Doha P.O. Box 26999, Qatar; mkarim@sidra.org; 6 College of Medicine, Qatar University, Doha P.O. Box 2713, Qatar; 7 Qatar Metabolic Institute, Hamad Medical Corporation, Doha P.O. Box 3050, Qatar; mramanjaneya@hamad.qa; 8 Translational Research Institute, Hamad Medical Corporation, Doha P.O. Box 3050, Qatar; 9 Department of Pediatrics, Weill Cornell Medicine, New York, NY 10021, USA; stw2006@med.cornell.edu; 10 Department of Pediatrics, Weill Cornell Medicine-Qatar, Doha P.O. Box 24144, Qatar

**Keywords:** children, asthma, obesity, sphingolipids, serine palmitoyltransferase

## Abstract

Asthma and obesity are two of the most common chronic conditions in children and adolescents. There is increasing evidence that sphingolipid metabolism is altered in childhood asthma and is linked to airway hyperreactivity. Dysregulated sphingolipid metabolism is also reported in obesity. However, the functional link between sphingolipid metabolism, asthma, and obesity is not completely understood. This paper describes the protocol of an ongoing study on sphingolipids that aims to examine the pathophysiology of sphingolipids in childhood asthma and obesity. In addition, this study aims to explore the novel biomarkers through a comprehensive multi-omics approach including genomics, genome-wide DNA methylation, RNA-Seq, microRNA (miRNA) profiling, lipidomics, metabolomics, and cytokine profiling. This is a cross-sectional study aiming to recruit 440 children from different groups: children with asthma and normal weight (*n* = 100), asthma with overweight or obesity (*n* = 100), overweight or obesity (*n* = 100), normal weight (*n* = 70), and siblings of asthmatic children with normal weight, overweight, or obesity (*n* = 70). These participants will be recruited from the pediatric pulmonology, pediatric endocrinology, and general pediatric outpatient clinics at Sidra Medicine, Doha, Qatar. Information will be obtained from self-reported questionnaires on asthma, quality of life, food frequency (FFQ), and a 3-day food diary that are completed by the children and their parents. Clinical measurements will include anthropometry, blood pressure, biochemistry, bioelectrical impedance, and pulmonary function tests. Blood samples will be obtained for sphingolipid analysis, serine palmitoyltransferase (SPT) assay, whole-genome sequencing (WGS), genome-wide DNA methylation study, RNA-Seq, miRNA profiling, metabolomics, lipidomics, and cytokine analysis. Group comparisons of continuous outcome variables will be carried out by a one-way analysis of variance or the Kruskal–Wallis test using an appropriate pairwise multiple comparison test. The chi-squared test or a Fisher’s exact test will be used to test the associations between categorical variables. Finally, multivariate analysis will be carried out to integrate the clinical data with multi-omics data. This study will help us to understand the role of dysregulated sphingolipid metabolism in obesity and asthma. In addition, the multi-omics data from the study will help to identify novel genetic and epigenetic signatures, inflammatory markers, and mechanistic pathways that link asthma and obesity in children. Furthermore, the integration of clinical and multi-omics data will help us to uncover the potential interactions between these diseases and to offer a new paradigm for the treatment of pediatric obesity-associated asthma.

## 1. Background

Asthma, a chronic condition marked by recurrent breathing difficulties, significantly impacts global health, especially among children [1,2], and ranks in the top 20 chronic diseases among children [2]. Influenced by a complex interplay of environmental and genetic factors, asthma affects over 300 million individuals worldwide, contributing to a considerable economic burden, particularly evident in low-income countries [3]. 

The prevalence of both asthma and obesity has increased globally in the past few decades. The disturbing simultaneous rise of asthma and obesity in children strongly suggests an interrelation between these conditions [4,5]. Children in Qatar are a valuable cohort to study this interaction, as the prevalence of asthma and obesity in this population is high [6,7,8]. 

Pertinently, systematic reviews and meta-analyses indicate that overweight and obese children face a substantially higher risk of developing asthma [4,5]. One review reported a 20% increase and a twofold increase in asthma’s prevalence in overweight and obese children, respectively [4], while another review reported a 20% and a 40% risk increment in overweight and obese children, respectively [5]. Obesity-related factors such as altered lung mechanics, hyperinsulinemia, increased inflammation, metabolic dysregulation, and microbiome dysbiosis are implicated in the pathogenesis of asthma [9,10]. 

This correlation necessitates comprehensive research focusing on the underlying mechanisms. One promising area of investigation is the role of sphingolipids, a class of lipids involved in maintaining the integrity of cell membranes, and which serve as signaling molecules in various cellular processes, including cell proliferation, differentiation, signal transduction, immune response, and apoptosis [11]. Genome-wide association studies (GWASs) correlate polymorphisms in chromosome 17q21 that lead to higher expression of Orosomucoid like 3 (ORMDL3), specifically in blood and airway smooth muscle [12,13,14,15,16,17]. 

**Figure 1:**
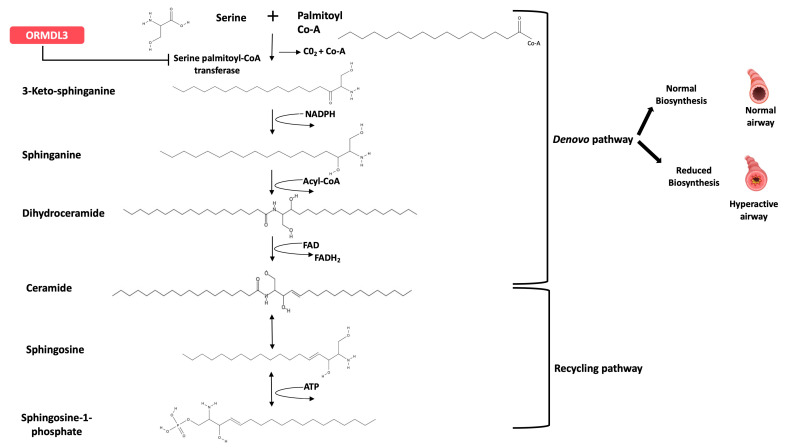
De novo and recycling pathways of sphingolipid biosynthesis: Enhanced expression of ORMDL3 inhibits the activity of the serine palmitoyltransferase (SPT) enzyme, leading to decreased de novo sphingolipid synthesis, consequently increasing airway hyperreactivity. Enhanced airway hyperresponsiveness is a major feature that promotes asthma’s pathogenesis.

ORMDL3 is a key regulator of sphingolipid synthesis [18,19,20,21,22,23,24,25] (Figure 1). The genotype-dependent effect on sphingolipid synthesis was demonstrated in a cohort of asthmatic children from New York using targeted blood sphingolipidomics and metabolic labeling of peripheral blood mononuclear cells (PBMCs) with the serine palmitoyltransferase (SPT) substrate, serine [26], supporting the longstanding hypothesis that sphingolipid synthesis is decreased in asthma [13,18,27], especially in children [28]. 

**Figure 2:**
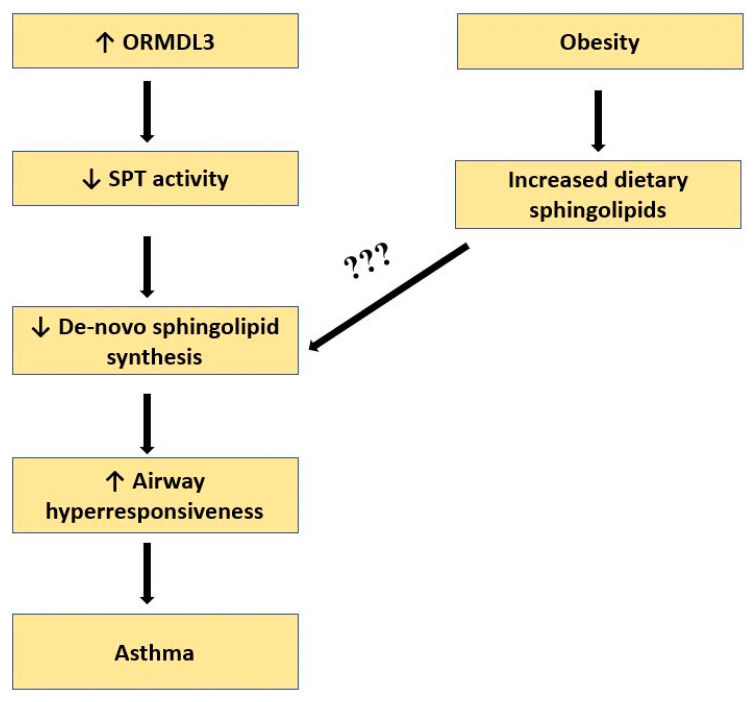
Potential role of obesity in dysregulating the de novo biosynthesis of sphingolipids. ↑: increase; ↓: decrease.

Mice with decreased de novo sphingolipid synthesis have increased airway reactivity [16]. This increased airway reactivity can also be reversed by specifically increasing sphingolipids that are exclusively generated by de novo sphingolipid synthesis. The exact mechanisms of how decreased de novo sphingolipid synthesis stimulates airway hyperreactivity are still under active investigation. Changes in magnesium homeostasis have been found in sphingolipid-deficient animals, and ORMDL3 has been shown to stimulate airway smooth muscle cell proliferation and calcium oscillations [16,29]. Studies in humans on sphingolipid metabolism in asthma and obesity have mostly analyzed the sphingolipid levels in blood. Interestingly, in children with asthma and asthma-associated 17q 21 genotypes, sphingolipid levels are increased in plasma but decreased in blood cells [26] Similarly, among sphingolipids, mainly ceramides, dihydroceramides, and sphingomyelins are increased in plasma or serum in obesity [30,31,32]. Dietary effects on sphingolipid synthesis could be of possible relevance in linking asthma to obesity, especially in individuals with genetic variations that affect sphingolipid synthesis (Figure 2). Possible similarities of obesity and asthma in sphingolipid metabolism warrant further investigation to understand whether there is a synergistic impact of dysregulated sphingolipid metabolism on asthma’s pathogenesis when both of these conditions coexist. 

Our cross-sectional study, known as the sphingolipids in obesity and asthma in pediatrics (SOAP) study, proposes a multifaceted approach to exploring this connection by examining sphingolipid profiles in a pediatric population afflicted with both asthma and obesity, seeking to uncover insights that could lead to improved therapeutic interventions. Central to our methodology is the integration of diverse datasets: we plan to compile and synthesize data from various domains, including clinical data (such as asthma severity and medication use), physiological data (like spirometry results and body mass index (BMI)), biochemical data (e.g., sphingolipid profile, cytokine profile, and metabolome of blood samples), and genetic data (e.g., whole-genome sequencing, RNA-Seq, and DNA methylation studies). This data integration will be achieved through a structured approach that correlates these different data types, allowing for a comprehensive analysis that accounts for the multifactorial nature of asthma and obesity. By employing advanced statistical methods and data integration techniques, we aim to identify patterns and interactions that might elude more traditional analyses. These findings could not only deepen our comprehension of these interlinked epidemics but also inspire new avenues for treatment and prevention. 

### Study Hypothesis and Aims

The overlying hypothesis of our study is that sphingolipid synthesis is affected in asthma and obesity in a way that leads to increased airway reactivity, with or without inflammation.

The current study aims to (1) analyze sphingolipids and their synthesis in children with asthma and obesity; (2) determine the genetic makeup, epigenetic signatures, transcriptomic signatures, miRNA profile, cytokine profile, and metabolomic and lipidomic signatures associated with asthma and obesity in children; (3) identify novel biomarkers associated with asthma and obesity that could be useful for diagnostic and therapeutic guidance; and (4) perform a multivariate analysis to integrate the multi-omics data and clinical data to understand the molecular mechanisms governing the disease pathology of asthma and obesity.

## 2. Methods

### 2.1. Study Design and Population

This is a cross-sectional study that commenced in August 2017 and will continue throughout December 2023. The study will include a cohort of 440 children from the Middle East and North Africa (MENA) region, residing in Qatar. The research will be conducted at Sidra Medicine, a private tertiary care hospital for children and women in Doha, Qatar.

The groups studied will be as follows: (1) diagnosed with asthma and normal weight (Group A), (2) diagnosed with asthma and overweight/obese (Group B), (3) asthma-free and overweight/obese (Group C), (4) asthma-free and normal weight (Group D), and (5) siblings of Groups A and B (Group E). All of the children who have asthma and are either normal weight or overweight or obese, along with their healthy siblings, will be recruited from the pediatric pulmonology outpatient clinic. The children with normal weight and no asthma will be recruited from the general pediatric outpatient clinic, where they are visiting for their immunization and general follow-up. Children who are overweight or obese will be recruited from the pediatric endocrinology department. After the child is shortlisted and screened by the clinical team, the child will be referred to a research coordinator to start the enrollment process. 

Asthma is diagnosed by the attending physician and confirmed using the International Study of Asthma and Allergies in Childhood (ISAAC) [33]. Children registered as asthmatic or non-asthmatic at Sidra Medicine’s outpatient clinic will be screened retrospectively for eligibility by the investigators through reviewing their medical records and laboratory results and shortlisting them, before approaching them as potential subjects.

Prior to recruitment and the consent process, the research coordinator will verify the eligible children according to BMI percentiles for age and gender. Children aged 6–10 years will be asked to provide verbal assent, and those who are aged 11 years and less than 18 years will be asked to provide a written signed assent form. The parents of all children enrolled will be asked to provide a signed informed consent form. The eligible children will be recruited as per the inclusion and exclusion criteria shown in Table 1.

**Figure 3:**
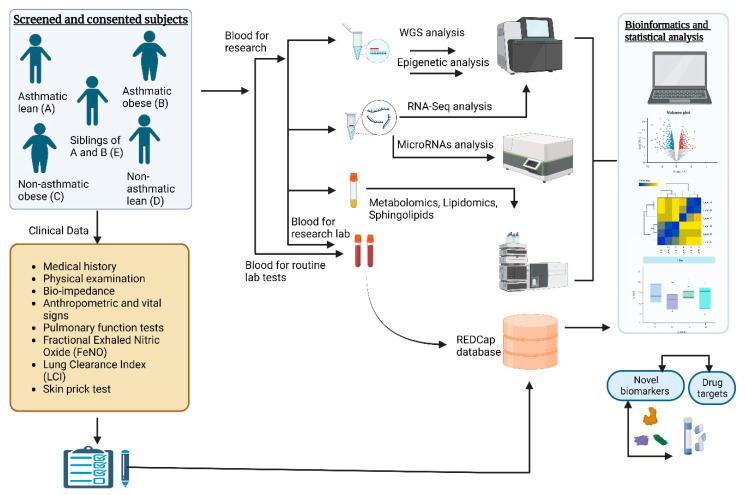
Flowchart of the summary of study protocol: clinical and biological measurements.

Children with severe allergies who cannot undergo skin allergy testing, based on clinical judgement, will be excluded from the allergy testing. However, they will be recruited, as the study aims to examine a range of asthma severity, including allergic and non-allergic cases. After providing the informed consent form, each child will undergo the routine and research procedures described in Table 2 and Figure 3.

### 2.2. Testing Procedures and Measurements

#### 2.2.1. Questionnaires (Self-Reported)

The SOAP questionnaire includes information on demographic data, family medical history, general health, medication, asthma, rhinitis, eczema and its management, cough and phlegm, breathlessness, consumption of food using a food frequency questionnaire, technology use, and body image perception. This information will be recorded directly by the participating children who are 13–17 years of age, while the data will be entered by parents/guardians in the case of children who are 6–12 years of age. In addition, information on birth data, diseases, immunizations, and home (living place) will also be obtained for the children aged 6–12 years.

The pediatric quality of life inventory (Peds QL) is a questionnaire with asthma and quality of life modules according to age-specific groups of 5–7 years old, 8–12 years old, and 13–17 years old. Each set of these questionnaires has two branches—one to be completed by the child, and the other by the parent or guardian.

A 3-day food diary will be used to record the food/drink intake of each subject over a period of three consecutive days, starting from the day of recruitment. This diary will include all meals, snacks, and special dietary supplements. The diary will be divided into time slots per day (e.g., breakfast, mid-morning, lunch, mid-afternoon, evening, and bedtime). The subjects will be encouraged to provide a detailed description of the food they consume, by providing household measurements (e.g., spoon, cup), specifying portion sizes or weights, reporting food brand names, and mentioning the method of cooking, if applicable.

#### 2.2.2. Medical History and Physical Examination

The medical history completed by the treating physician will include the child’s history of asthma, eczema, allergies, rhinitis/hay fever, diabetes mellitus, hypertension, cholesterol, cancer, heart disease, liver problems, thyroid, sleep issues, obesity, and other medical conditions. 

A comprehensive physical examination will be carried out by the treating physician. This will include a general examination, chest examination, cardiovascular (CVS) assessment, abdominal examination, inspection of the skin, examinations of the mouth, nose, and ears, neck examination, and eye check. All systems or organs will be reported as normal, abnormal, or not examined. Additional details on chest examinations will be reported in the event of any abnormalities, such as wheezing, crepitations, chest abnormalities, symmetrical movement, or retractions.

#### 2.2.3. Anthropometry

All anthropometric measurements will be performed by trained health professionals at the clinic. Height (cm), weight (kg), waist circumference (cm), hip circumference (cm), neck circumference (cm), and chest circumference (cm) will be measured using a standardized protocol. Weight and height will be measured using a digital scale with a stadiometer (Seca 284, Hamburg, Germany). All anthropometric measurements will be recorded to the nearest 0.1 unit. BMI is defined as weight (kg) divided by the square of height (in meters) (kg/m^2^). Overweight and obesity are defined as BMI above the 85th percentile and BMI above the 95th percentile for age and sex, respectively [34]. Three consecutive measures of anthropometric measurements will be taken, and the average values will be used in the analysis.

#### 2.2.4. Vital Signs

Blood pressure (systolic/diastolic), heart rate, respiratory rate, and body temperature (oral) will be measured by the treating physician as per the standard operating procedures. Blood pressure (BP) will be measured using a digital sphygmomanometer (Welch Allyn Connex Integrated Wall System, New York, NY, USA) in the sitting position after five minutes at rest. All vital signs will be measured thrice consecutively, and the average of the three readings will be used in the analysis. 

#### 2.2.5. Bioimpedance

Bioimpedance measures will be obtained using a body composition analyzer (Tanita BC-420MA, Tokyo, Japan) for all of the children in the different study groups, including BMI (kg/m^2^), fat percentage (%), fat mass (kg), free fat mass (kg), total body water (kg), and basal metabolic rate (BMR) (kcal/day).

#### 2.2.6. Glucose, Insulin, Lipids, and Other Blood Tests

Plasma fasting glucose and 120-min glucose concentrations will be measured using a hexokinase photometric assay, serum lipid concentrations will be measured by photometric assay with a DxC 700 AU Chemistry Analyzer, and fasting insulin will be measured by immunoassay (Beckman Coulter, Brea, CA, USA). The HOMA-IR index will be calculated by multiplying the plasma glucose (mmol/L) by the fasting serum insulin (μU/L) and dividing by 22.5 [35], which is validated in children and adolescents [36,37]. Complete blood counts, biochemistry, vitamin D, and thyroid-stimulating hormone (TSH) will be obtained for all subjects, while HbA1c and C-peptide will be measured for obese (asthmatic and non-asthmatic) and normal-weight asthmatic children. 

#### 2.2.7. Pulmonary Function and Plethysmography Tests

Lung function tests will be carried out using spirometry and the bronchodilator test (Jaeger MS-PFT Analyzer, CareFusion, Leibnizstrasse, Germany). The tests will include forced vital capacity (FVC), forced expiratory volume in 1 s (FEV1), FEV1/ FVC, forced expiratory flow at 25% and 75% of the pulmonary volume (FEF), peak expiratory flow (PEF), peak inspiratory flow (PIF), and forced expiratory time (FET). Plethysmography (Jaeger MS-PFT Analyzer, CareFusion, Leibnizstrasse, Germany) tests will be performed to measure specific airway resistance (sRAW), vital capacity (VC), inspiratory capacity (IC), functional residual capacity (FRCpleth), expiratory reserve volume (ERV), residual volume (RV), total lung capacity (TLC), and RV/TLC. All evaluations will be performed by professionally trained pulmonologists.

#### 2.2.8. Bronchodilator Response

After the baseline spirometry, children will be given 400 μg (4 puffs) of salbutamol. Spirometry will be repeated 15 min after salbutamol administration. A significant response to the bronchodilator will be identified if baseline FEV1 is increased by ≥12% after the bronchodilator administration. 

#### 2.2.9. Fractional Exhaled Nitric Oxide (FeNO)

The concentration of nitric oxide (NO) will be measured using the chemiluminescence method (Ecomedic’s Exhalyzer CLD 88, Duernten, Switzerland): the child will inhale NO-free air from the device and then exhale, aiming to achieve a constant expiratory flow of 50 mL/s until achieving a plateau of nitric oxide concentration. The measurements will be repeated until either two repeatable values are within 5% of one another or three values are within 10% of one another, which will be reported as an average in the analysis.

#### 2.2.10. Lung Clearance Index (LCI)

The LCI will be measured using the N_2_ multiple-breath washout technique (Ecomedic’s Exhalyzer CLD 88, Duernten, Switzerland). Oxygen (O_2_) and carbon dioxide (CO_2_) will be measured during the testing. The nitrogen (N_2_) will be indirectly measured using Dalton’s law of partial pressures. The N_2_ washout test involves getting the patient to breathe normally through the mouthpiece, breathing 100% oxygen until the exhaled nitrogen concentration falls to or below 1/40th of its starting concentration for 3 consecutive breaths. The average test result will be accepted if the two trials are within 5% or three trials are within 10% deviation. 

#### 2.2.11. Allergy Test

Allergen-specific serum IgE antibody levels will be measured by fluorescent enzyme immunoassay (FEIA) using two Phadia 250 analyzers (Thermo Fisher Scientific, Uppsala, Sweden), following the standard operating procedure developed in accordance with the manufacturer’s recommendations. A region-specific custom allergen panel will be designed to reflect the most relevant local food and aeroallergen sensitivities. For each child in the study, allergen-specific IgE responses to 5 allergen mixes and 18 individual allergens will be measured, comprising a potential maximum of 45 aero- and food allergens. Specific IgE results ≤ 0.34 kUA/L (as per Phadia’s established ranges) in response to allergen mixes will be considered negative to all individual allergens contained within the corresponding mix. A value of IgE ≥ 0.35 kUA/L will be defined as positive for the sensitization of a given child to an allergen mix, or to an individual allergen. For the positive allergen mixes, such as FX1 nut mix, MX2 mold mix, and FX5 food mix, specific IgE antibodies to relevant individual allergens will be routinely measured. The total IgE level in each sample will be measured by FEIA on the Phadia 250 analyzer according to the manufacturer’s recommendations.

#### 2.2.12. Allergy Skin Prick Test

The allergy skin prick test will be carried out using an allergen panel (AllerGen Medikal, Turkiye). After pricking the skin, 15 min will be allowed for any localized reaction to develop, after which the skin will be examined, looking for any positive reaction in the form a raised wheal surrounded by a red flare. Using a ruler, the wheal and flare sizes will be measured. Any allergen producing a wheal of ≥3 mm will be identified as positive, while others will be identified as negative as long as histamine produced a positive reaction and the negative control produced no reaction. 

#### 2.2.13. Cytokine and Adipokine Measurements

The levels of cytokines (IL-22, IFN-γ, TNFα, IL-2, IL-4, IL-5, IL-10, IL13, IL-17A, and IL-33) will be measured using a high-sensitivity multiplex kit (Merck Millipore, Burlington, MA, USA). Leptin will be measured using the Bio-Plex Pro Human Diabetes single-plex assay, and thymic stromal lymphopoietin (TSLP) will be measured using the Bio-Plex single-plex assay (Bio-Rad, Hertfordshire, UK).

#### 2.2.14. Sphingolipids and SPT Activity

The concentrations of different sphingolipids will be assessed in blood pellets and plasma derived from 4 mL of heparinized blood. The SPT assay will be carried out using PBMCs isolated from 8 mL of blood using cell preparation tubes (CPT). Targeted analysis of key metabolites of sphingolipid groups allows for the comprehensive profiling of the de novo synthesis and recycling pathway. As sphingolipids contain long-chain hydrocarbon groups and have hydrophobic and hydrophilic properties [38], high-pressure liquid chromatography coupled with tandem mass spectrometry (HPLC-MS/MS) is used for their analysis [39,40,41,42]. Studies with human blood have established the methodology, normal value ranges, and optimal sample conditions for the proposed analyses in the blood of children with asthma [26,40,42,43,44]. Briefly, a sample will be extracted with dichloromethane:methanol (1:1, *v*/*v*), followed by centrifugation. The supernatant will be evaporated and reconstituted in methanol for injection into the chromatographic system. A gradient mobile phase consisting of acetonitrile, water, isopropanol, and formic acid will be applied to the chromatographic column (InfinityLab Poroshell 120 EC-C18, 2.1 × 50 mm, 2.7 µm, Agilent Technologies, Santa Clara, CA, USA). The parameters of the electrospray ionization (ESI)–triple-quadrupole mass spectrometer will be optimized with pure standards of sphingolipids (Avanti Polar Lipids, Pelham, AL, USA) and operated in positive ionization multiple reaction mode. Mixed standards prepared from the accurate weighing of pure sphingolipid materials will be assayed alongside the whole-blood samples to obtain quantitative results of the samples by volume. 

To assess cellular sphingolipid synthesis as a surrogate of SPT activity, a stable isotope-based cell culture method [45] will be used to measure the incorporation of the SPT substrate, serine, into sphinganine in PBMCs [26]. This robust, non-radioactive method requires only a small volume of blood, little pre-analytical handling, and a short incubation time. PBMCs isolated by Ficoll gradient will be incubated for 30 min at 37 °C, with the stable isotope L-[U-^13^C,^15^N]-serine subsequently incorporated into sphinganine. 

#### 2.2.15. Metabolomics and Lipidomics

Samples will be prepared by protein precipitation or liquid–liquid extraction, and the supernatant will be evaporated, reconstituted, and injected on an ultrahigh-pressure liquid chromatograph with an Orbitrap Fusion Lumos Tribrid mass spectrometer (Thermo Scientific, Waltham, MA, USA). A portion of all samples will be mixed to create an identification (ID) sample. Each sample will be acquired at 240K mass resolution in separate ESI-positive and -negative polarity modes using gradient mobile phases. The high mass resolution will allow for fine isotopic measurements that can be used to determine the elemental composition of the metabolites. The ID samples will also be subjected to MS2 fragmentation to aid in metabolite annotation. Data processing for retention time alignment, normalization, and feature detection will be conducted, and an in-house library and online databases will be utilized to annotate the lipids and metabolites. The peak abundance of each annotated lipid or metabolite in each sample will be used for statistical and pathway analysis after data transformation and Pareto scaling.

#### 2.2.16. DNA Extraction

Peripheral whole-blood samples will be processed for DNA isolation using the automated QIASymphony SP instrument according to the QIAamp DNA Midi Kit protocol’s recommendations. The DNA quality and quantity assessment will be carried out using a NanoDrop 8000 (Thermo Fisher Scientific, Waltham, MA, USA). The absorbance at 260 and 280 nm wavelengths will be assessed to check the DNA purity. The extracted DNA will be processed for whole genome sequencing WGS and Illumina Infinium EPIC methylation profiling.

#### 2.2.17. Whole-Genome Library Construction and Sequencing

DNA will be quantified using the Quant-iT dsDNA Assay (Invitrogen, Waltham, MA, USA) on the FlexStation 3 (Molecular Devices, Sunnyvale, CA, USA). The library will be constructed from 250 ng of DNA with the Illumina TruSeq DNA Nano kit and amplified for 8 cycles following ligation with IDT for Illumina–TruSeq DNA UD Index adapters. The library quality and concentration will be assessed using the LabChip NGS 3K assay (PerkinElmer, Waltham, MA, USA) on a PerkinElmer GX2, and qPCR will be performed using the KAPA Library Quantification Kit (Roche, Pleasanton, CA, USA) on a Roche LightCycler 480 II. Genomic libraries will be sequenced with paired-end 150 bp on the Novaseq 6000 system (Illumina, San Diego, CA, USA), using the manufacturer’s recommended protocol to achieve a minimum average coverage of 30X [46].

#### 2.2.18. Illumina EPIC Methylation

Genomic DNA will be bisulfite-converted using the EZ DNA Methylation Kit (Zymo Research, Irvine, CA, USA). The genome-wide methylation will be assessed using the Infinium Human Methylation EPIC BeadChip Kit (Illumina, San Diego, CA, USA). The EPIC BeadChips cover ~850k human CpG loci at a single-nucleotide resolution. The single-base extension reaction will be carried out on the Freedom Evo 150 automation system (Tecan Group Ltd., Männedorf, Switzerland). The arrays will be scanned on the Illumina iScan platform. Methylated and unmethylated signal intensity data will be generated as idat. files on the Illumina GenomeStudio software (Illumina, San Diego, CA, USA) and background-normalized using negative control probes. The QC of the assay control probes will be performed on GenomeStudio (Illumina, San Diego, CA, USA).

#### 2.2.19. RNA Extraction

The blood collected in PaxGene RNA tubes will be stored at −80 °C until processing. The blood tubes will first be allowed to thaw overnight before RNA purification and then centrifuged for 10 min at 5000× *g*, using a swing-out rotor to collect the pellet. After decanting the supernatant, the pellet will be resuspended according to the manufacturer’s instructions. The tubes will then be transferred to the QIASymphony SP system for automated RNA isolation, following the manufacturer’s recommendations. The absorbance at 260 and 280 nm wavelengths will be assessed on a NanoDrop 8000 spectrophotometer (Thermo Fisher Scientific, Waltham, MA, USA) to check the RNA purity and quantity. The total RNA extracted will be processed for total RNA sequencing and miRNA profiling.

#### 2.2.20. Total RNA Library Construction and Sequencing

After the extraction of total RNA from human whole blood, the sample integrity and concentration will be controlled and measured using the standard sensitivity RNA assay on the PerkinElmer Caliper LabChip GXII. Then, 500 ng of total RNA will be used for library preparation using the Illumina-TruSeq Stranded Total RNA Globin Kit, which assists in depleting ribosomal RNA (rRNA) and globin-encoding mRNA using biotinylated oligos combined with streptavidin-coated magnetic beads. The cDNA obtained after reverse transcription using Superscript IV will be ligated with IDT for Illumina-TruSeq RNA UD Index adapters and amplified for 10 cycles. The library quality and concentration will then be assessed using the LabChip NGS 3K assay (PerkinElmer, Waltham, MA, USA) on a PerkinElmer GX2, and by qPCR using the KAPA Library Quantification Kit (Roche, Pleasanton, CA, USA) on a Roche LightCycler 480 II. The RNA libraries will be sequenced with paired-end 150 bp reads on the Novaseq 6000 system (Illumina, San Diego, CA, USA), using the manufacturer’s recommended protocol.

#### 2.2.21. miRNA Profiling

The total RNA will be processed for miRNA expression profiling using NanoString (NanoString Tech Inc., Seattle, WA, USA). Sample preparation will involve a multiplexed annealing of specific tags to their target miRNAs, a ligation reaction, and an enzymatic purification to remove the unligated tags, following the protocol recommendations (Human v3 miRNA assay). The samples will be run on the nCounter FLEX system. The digital images will be processed on the nCounter Digital Analyzer, and the barcode counts will be tabulated in Excel format. The QC analysis will be performed on the nSolver software, v4.0 (NanoString Tech Inc., Seattle, WA, USA). Raw counts will be normalized using internal positive controls as well as reference genes provided in the codeset. Background noise will be calculated using non-human negative control probes. 

### 2.3. Sample Size Calculation and Statistical Analysis

The sample size was calculated based on our pilot data examining the serum ceramide/dihydroceramide ratio in children. We required 90 subjects per group to detect a difference in the mean ratios of 0.25 (unitless ratio) between non-atopic asthmatic (mean 1.47) and non-atopic and non-asthmatic (mean 1.22) children, with a standard deviation of 0.6 at 80% power and a 5% level of significance. A recent study by Ono et al. [26] in children (60 per group) was able to detect blood sphingolipid differences between asthma and non-asthma, including 17q21 genotype-specific differences, so a size of 90 per group in our study would be sufficient to detect sphingolipid differences between the groups.

Demographic and clinical characteristics will be summarized as means and standard deviations for normally distributed data, medians and interquartile ranges for skewed data, and frequencies and percentages for categorical data. Group comparisons will be carried out using an analysis of variance (ANOVA) or the Kruskal–Wallis test to identify differences in the clinical and sphingolipid variables, adjusting for age and gender. Analyses will be conducted if there are differences in sphingolipids between the asthma and control groups by gender and stratified by total IgE (low IgE: <200 IU/mL, high IgE: ≥200 IU/mL). The chi-squared test will be used for testing the associations between categorical variables. A regression analysis will be carried out with adjustment for potential confounding factors. For sphingolipids, a principal component analysis will be carried out to identify patterns that are associated with obesity and asthma. Appropriate adjustments will be made for multiple comparisons, thus avoiding type 2 errors. For skewed data, appropriate transformations will be applied and, if not possible, non-parametric tests will be used. 

The data from whole-genome sequencing will be used to examine the genetic variants associated with asthma and obesity using the Genome Analysis Toolkit (GATK)’s best practices pipeline [46]. The distinctive gene expression patterns and miRNA profiles between the obese asthmatic and normal-weight asthmatic groups will be derived using different packages and software, such as DESeq2 and Cytoscape [47,48,49]. Furthermore, the ChAMP and minfi Bioconductor packages will be used to identify differentially methylated regions (DMRs) and individual CpG sites (DMPs) [50,51,52]. The genomic annotation utilized to detect DMRs and DMPs will be based on the human genome assembly GRCh38/hg38. For metabolomic and lipidomic data, we will conduct univariate and multivariate analyses. These analyses will include the use of support-vector machines (SVMs), partial least squares discriminant analysis (PLS-DA), and random forests. Receiver operating characteristic curve (ROC curve) analyses will be performed to identify key features (e.g., metabolites and lipids) capable of distinguishing between the asthmatic groups.

The associations between genotypes of asthma at 17q21, obesity, and levels of sphingolipids will be examined using Spearman’s rank correlation and Kruskal–Wallis one-way analysis of variance. All downstream analyses will be carried out using R statistical software and appropriate Bioconductor tools.

### 2.4. Data Integration

When a study participant is recruited, a unique identification number (ID) is provided. All case report forms (CRFs) including demographic, anthropometric, and pulmonary function data will be entered into Research Electronic Data Capture (RedCap) software (Vanderbilt University, Nashville, TN, USA) routinely as and when the data are collected, on a real-time basis. These data will be entered twice into RedCap by different research personnel for verification purposes. The lab data, such as blood analysis and medications, will be extracted from CERNER by the programmer using the date of recruitment, hospital number, and participant research ID, before being exported to RedCap and verified for quality and accuracy. These data from RedCap will be exported to standard statistical software, the STATA (Version SE/17, StataCorp LLC, College Station, TX, USA) and then further checked for missingness, range, consistency, and outliers.

Integrating multi-omics data will involve a systematic process to harness the collective power of diverse omics datasets, using scheduled batches that we generate in this study. The process will begin with preprocessing with standardization to ensure consistency, where data will be gathered from different sources, such as sphingolipid analysis, whole-genome sequencing, transcriptome analysis, DNA methylation study, metabolomics, and miRNA analysis. Subsequently, significant features will be selected from single-nucleotide polymorphisms, differentially expressed genes, methylation positions, metabolites, lipids, and miRNAs within each dataset. These individual multi-omics databases will be merged with the master database with the unique ID numbers of the participants using STATA software. The integrated master database will be used for multivariable analysis to visualize patterns and relationships, and the results will be interpreted with biologically meaningful insights (Figure 3). 

## 3. Expected Results and Outcomes

Based on the previous findings by Ono et al. and Rago et al., we anticipate differences in the levels of sphingolipids and altered SPT activity between the different asthma endotypes of our study [26,53]. Correlating metabolites with genotype status has become an attractive strategy in metabolomics studies. We plan to correlate sphingolipid levels with the most common asthma- and obesity-related single-nucleotide polymorphisms in our cohort, which will help us to further classify our asthma patients and understand the molecular mechanisms of the disease’s pathogenesis. Based on previous findings in the literature and the observed differences in the clinical features between normal-weight asthma and obese asthma in our pediatric cohort, we expect to identify distinct genetic variations and DNA methylation signatures, altered gene expression profiles, differences in cytokine levels, and altered metabolomic and lipidomic signatures that are associated with the specific genotypes and phenotypes of our cohort [54,55,56].

To the best of our knowledge, this is the first comprehensive clinical study on sphingolipid synthesis that will examine its links to asthma and obesity in children in Qatar. The findings of our study will help to evaluate sphingolipids’ composition and to analyze the effects of decreased de novo sphingolipid synthesis in children with and without asthma and obesity. Furthermore, the results of the multi-omics study will provide valuable information about the variations in genetic makeup, gene expression, miRNA profile, cytokine levels, and metabolomic and lipidomic profiles across different asthma endotypes. The study will also use multi-omics data and clinical parameters to develop a model to classify different asthma endotypes. The identified biomarkers and molecular mechanisms will serve as a benchmark for future functional studies and the development of personalized therapies. This study is expected to contribute significantly to the understanding and treatment of asthma and obesity in pediatric populations in Qatar. In addition, the databases containing the clinical and operational data sources that will be developed as part of this study can be potentially used for health decision-making and future research in asthma and chronic obstructive pulmonary disease [57,58]. 

## 4. Strengths and Limitations

➢ The study will provide comprehensive genetic, epigenetic, metabolomic, and lipidomic data that affect sphingolipid metabolism to characterize endotypes of children with asthma and obesity, which will be critical to understand the underlying pathophysiology and to develop optimal treatment strategies.
➢ The study will use harmonized procedures for measuring anthropometry, lung function, biochemical risk factors, sphingolipid synthesis, and multi-omics data.
➢ The study will include a nested family group of healthy siblings of asthmatic children to assess environmental and genetic factors associated with asthma and obesity.
➢ For the assessment of sphingolipids, we will rely on measurements of blood, since it cannot be measured noninvasively in the airways of children with asthma. Whole-blood sphingolipids have been shown to be correlated with asthma in children.
➢ Body mass index (BMI) will be used to categorize overweight and obese children, as it can be easily assessed at the time of recruitment, with the caveat that BMI does not distinguish either subcutaneous fat from visceral adiposity or fat mass from lean mass.

## Figures and Tables

**Table 1:** Inclusion and exclusion criteria.

Inclusion Criteria	Exclusion Criteria
➢ Aged 6 to 17 years from the Middle East and North Africa region ➢ Diagnosed with asthma and normal weight (Group A) ➢ Diagnosed with asthma and overweight or obese (Group B) ➢ Overweight or obese (Group C) ➢ Normal weight (Group D) ➢ Siblings of Groups A and B (Group E)	➢ Non-asthma chronic lung disease ➢ Bronchopulmonary dysplasia (BPD) ➢ Syndromic disorders, e.g., Down’s syndrome, Turner’s syndrome ➢ Inborn errors of metabolism ➢ Symptomatic or previously symptomatic congenital heart disease ➢ Craniofacial abnormalities ➢ Primary thoracic cage abnormalities ➢ Neuromuscular disorders ➢ Swallowing disorders ➢ Secondary endocrinopathies causing obesity ➢ Ongoing treatment for cancer

**Table 2:** Summary of the SOAP study protocol test procedures.

Schedule of Assessments, Tests, and Procedures		Group A	Group B	Group C	Group D	Group E
		Asthma and Normal Weight	Asthma and Overweight or Obese	Obese or Overweight and No Asthma	Normal Weight and No Asthma	Siblings of Groups A and B
Demographics and Medical History		◎	◎	◎	◉	◉
Questionnaires SOAP questionnaire (age 6–12 years, 13–17 years) Peds QL (asthma and quality of life modules (ages 5–7, 8–12, and 13–17 years) Food frequency questionnaire (FFQ) and 3-day food diary		◉	◉	◉	◉	◉
Anthropometry (height, weight, NC, CC, WC, HC)		◎	◎	◎	◉	◉
Physical examination (blood pressure (systolic/diastolic), heart rate, respiratory rate, body temperature)		◎	◎	◎	◉	◉
Bioimpedance measures (BMI, fat %, fat mass, free fat mass (FFT), total body water (TBW), basal metabolic rate (BMR))		◉	◉	◉	◉	◉
Pulmonary function tests (PFTs) Spirometry (FVC, FEV1, FEV1/ FVC, FEF 25–75%, PEF, PIF, FET) Plethysmography (sRAW, VC, IC, FRCpleth, ERV, RV, TLC, RV/TLC)		◎	◎	◉	◉	◉
Bronchodilator response		◎	◎			
Fractional exhaled nitric oxide (FeNO)		◎	◎	◉	◉	◉
Lung clearance index (LCI)		◎	◎	◉	◉	◉
Allergy test (skin prick)		◎	◎			
Blood tests						
FBC, biochemistry, vitamin D, TSH, FT4		◎	◎	◎	◎	◎
Allergy test		◎	◎			
HbA1c, insulin, C-peptide		◉	◉	◎		
Oral glucose tolerance test (OGTT), HOMA-IR, lipid profile			◉	◎		
Biological samples (fasted for at least 2 h)						
Blood	Sphingolipids, SPT assay, lipidomics, metabolomics, cytokines, whole-genome sequencing, DNA methylation, RNA-Seq, miRNA	◉	◉	◉	◉	◉

Note: ◎ indicates tests routinely performed for subjects during their clinical visits; ◉ indicates tests conducted for research purposes only. SOAP: sphingolipids in childhood obesity and asthma; NC: neck circumference; CC: chest circumference; WC: waist circumference; HC: head circumference; BMI: body mass index; FVC: forced vital capacity; FEV1: forced expiratory volume in 1 s, FEF 25–75%: forced mid-expiratory flow; PEF: peak expiratory flow; PIF: peak inspiratory flow, FET: forced expiratory time; sRAW: specific airway resistance; VC: vital capacity; IC: inspiratory capacity, FRCpleth: functional residual capacity measured by plethysmography; ERV: expiratory reserve volume; RV: residual volume; TLC: total lung capacity; FBC: full blood count; TSH: thyroid-stimulating hormone: FT4: free thyroxine test; HbA1c: glycated hemoglobin; C-peptide: connecting peptide; HOMA-IR; homeostatic model assessment for insulin resistance; SPT: serine palmitoyltransferase; DNA: deoxyribonucleic acid; RNA: ribonucleic acid.

## Data Availability

Not applicable.
